# 

**Published:** 1896-02

**Authors:** 


ESTABLISHED 1855. SUBSCRIPTION, $2.00.

### ATLANTA MEDICAL AND SURGICAL JOURNAL.

VOLUME XII. NEW SERIES. ATLANTA, GA., FEBRUARY, 1896. No. 12.

